# Editorial: Microelectronic Implants for Central and Peripheral Nervous System: Overview of Circuit and System Technology

**DOI:** 10.3389/fnins.2021.794944

**Published:** 2021-11-19

**Authors:** Alexandre Schmid, Takashi Tokuda, Ming-Dou Ker

**Affiliations:** ^1^Biomedical and Neuromorphic Microelectronic Systems BNMS, SCI-STI-AXS Station 11, Swiss Federal Institute of Technology EPFL, Lausanne, Switzerland; ^2^Institute of Innovative Research (IIR), Tokyo Institute of Technology (Tokyo Tech), Tokyo, Japan; ^3^Biomedical Electronics Translational Research Center, National Yang Ming Chiao Tung University, Hsinchu, Taiwan

**Keywords:** microelectronic implants, neuro-technologies, biomedical circuits and systems, neuroprosthetic systems, bioelectronics

## 1. Introduction

Research on fundamental properties and collaborative operation of neo-cortical microcircuitry has accelerated in the recent years, driven by the conviction that a deeper understanding of human brain will open the way to new medical techniques. Microelectronics has reached a state of maturity, where a single chip provides large amounts of computing power. Recent attempts in combining these two fields have successfully demonstrated some form of responsiveness between microelectronic circuitry and living matter. Novel microelectronic systems are developed as a major component of neurotechnolgy aiming at the closed-loop control of tissues or organs suffering functional malfunction or disease, the restoration of lost limb or organ functionality or bidirectional information passing and interpreting to increase human or amputee capability.

Among several contributors to neurotechnology, microelectronics plays a fundamental role that is evidenced by the emergence of multiple devices, circuit techniques and commercial products. Still, the domain is extremely dynamic and novelty is required to accommodate the usage of new fabrication technologies, the autonomous operation of implantable systems at extremely low power consumption, handling large amounts of data produced by modern and future massively multichannel bio-sensing devices, the extension toward new application fields in the form of therapeutic systems of newly considered diseases or disease prophylaxis. Furthermore, the widening usage of bio-medical and neuroprosthetic systems may result into the emergence of new challenges including the reliability of microelectronics, safety of produced data, invasiveness reduction that should also be tackled at the level of the new circuits and systems.

This Research Topic aims to gather results in recent neurotechnology in the form of a collection of review papers, and also present novel methods with high potential of creating the next generation of technology as an overview.

The focus of this Research Topic relates to ASIC/SoC and microelectronic circuit and system techniques that are applied to the development of advanced implantable bio-electronic and bio-medical systems, covering but not limited to low-power analog front-end interfaces for recording and stimulation, digital systems aiming at signal processing, feature extraction and pattern detection, power and data telemetry, enhanced ASIC fabrications aiming at new electrical or optical sensing and stimulating, full neuro-prosthetic systems and applications.

A set of ten papers have been selected to appear in this Research Topic on the basis of a thorough peer review process with iterations of manuscript revisions. The manuscripts have been assigned to four major subtopics, being understood that some papers contribute to several sub-topics, as detailed in the following.

## 2. Recording Circuits Techniques

Implantable recording devices are dedicated and complex systems designed to optimally capture the features of biological signals that are of importance to the subsequent analysis prescribed by the application, research or therapy. Consequently, all methods and circuits that are developed are specific in terms of the electrical specifications that are satisfied. Some techniques are commonly agreed upon to trade-off the bandwidth, gain and power and electronic noise of low-noise front-end amplifiers including multistage amplification, transistor sizing as well as the implementation of signal processing as analog modulation (Bagheri et al., 2017; Luo et al., 2019) for instance. Nevertheless, due to the very different nature of the signals to be recorded, the architecture and designs are not uniform.

Two issues of future implanted recorded systems have been emerging, namely their possible implementation using very-deep submicron fabrication technologies, and their necessary adaptations to support massively large numbers of input channels.

Noshahr et al. present a paper titled “Low-Cutoff Frequency Reduction in Neural Amplifiers: Analysis and Implementation in CMOS 65 nm” and discuss methods aiming at enabling the development of front-end ac-coupled amplifiers in deep-submicron fabrication technologies. Whereas, several bio-signals carry information contents in relatively low frequencies, modern submicron technologies tend to shift the low-corner cut-off frequency of amplifiers to higher values. The reasons of this behavior is identified and modeled and two possible solutions aiming at keeping the low-cutoff frequency low without increasing the circuit size are presented and measured.

Perez-Prieto et al. discuss in their paper titled “Recording Strategies for High Channel Count, Densely Spaced Microelectrode Arrays” many electrical issues that arise in the development of multi-channel recording implantable circuits. A classification of neural recording architectures depending on the position of the analog multiplexer in the signal path is presented. Specifically, time-division multiplexing is systematically analyzed as a suitable technique for analog front-end of implantable biomedical devices. The major architectures of time-division multiplexing at the analog front-end are presented and discussed.

## 3. Stimulation Circuits Techniques

The electrical stimulation of tissues is a proven technique which is the core of several therapeutic devices. Deep-brain and peripheral nerve electrical stimulation is applied in the control of neurological disorders including Parkinson's disease, epilepsy (Rolston et al., 2012).

Wu et al. present in their paper titled “Directions of Deep Brain Stimulation for Epilepsy and Parkinson's Disease” a systematic review of deep-brain stimulation systems aiming at controlling epilepsy and Parkinson's disease. Commercial devices operating in open and closed-loop are presented. Techniques pertaining to recording and stimulation and signal processing are discussed, and an overview of future prospects is presented.

Culaclii et al. discuss the necessity of controlling over arbitrary stimulation waveforms and present a multi-layer system that efficiently generates such stimuli in their paper titled “A Biomimetic, SoC-Based Neural Stimulator for Novel Arbitrary-Waveform Stimulation Protocols.” A Specific architecture involving three domains of devices is used to support the control. The system hardware is developed in an implantable version using some discrete components. The system is compatible with neural implants and supports a variety of irregular stimulation waveforms.

Finally, a fully implantable stimulator with sealed packaging and wireless data and power transmission is presented by Jiang et al. in their paper titled “A Versatile Hermetically Sealed Microelectronic Implant for Peripheral Nerve Stimulation Applications.” Electrical and in-field characterization results are presented. An architecture consisting of a wearable external station and an implantable stimulator is presented. Three stimulator chips are used that deliver up to 1 mA and 3 mA biphasic pulses to 12 different electrode configurations each. A high-voltage 600 nm CMOS fabrication technology is selected.

## 4. Systems With Closed-Loop Operation

Closed-loop implanted systems have naturally followed the successful development of individual blocks related to recording and stimulation. The decision to deliver electrical stimulation is formed from the some signal processing of the recorded brain signal data, on-chip, hence closing a loop consisting of the disabled tissue and the implanted autonomous control system.

Li et al. present a paper titled “Advances in Neural Recording and Stimulation Integrated Circuits” and discuss the different blocks of a closed-loop implant focusing on a state-of-the-art of implementation circuit techniques and the key issues that each technique aims at solving. An introduction to the basics of neuro-prosthetic systems is presented. The technology, architecture and key issues of neural recorders is discussed. Considerations related to neural recording and stimulation and future trends are proposed.

Additional modules related to artifact removal in signal processing as well as wireless data and power transmission are discussed by Cho et al. in their paper titled “Energy-Efficient Integrated Circuit Solutions Toward Miniaturized Closed-Loop Neural Interface Systems.” A review of state-of-the-art of implantable stimulators is presented along with a discussion of recording systems and the requirement for implantable closed-loop electrical systems.

Stimulation in the optical domain is a recent technology with respect to electrical stimulation, that aims at optogenetics, i.e., the selective optical stimulation of genetically modified cells. A wireless platform aiming at closed-loop optogenetic control in freely moving rodents is presented in the paper titled “A Wireless Electro-Optic Platform for Multimodal Electrophysiology and Optogenetics in Freely Moving Rodents” by Bilodeau et al.. A data reduction rate of 7.77 is achieved by applying real-time compression. The systems records from up to 32 channels and stimulates into four optical channels. Off-the-shelf and FPGA components are used in the development, while signal processing algorithms are embedded to separate action potentials, low field potentials and electromyography signals. The full systems weights 4.7 g and includes a 100 mAh battery.

## 5. Systems With Imaging Capability

Brain imaging has been considered for the sake of studying the activity and operation of some regions. Though, bulky systems based on microscopy were used that limit the freedom of the animal under observation and thus also alter the results. Implantable brain imagers are new systems aiming at disclosing their results obtained in real life conditions, and open a new field of research.

Pakpuwadon et al. present a paper titled “Self-Reset Image Sensor With a Signal-to-Noise Ratio Over 70 dB and Its Application to Brain Surface Imaging” in which a high-dynamic range CMOS sensor is proposed as a suitable solution to detect very small signals. A self-resetting photodiode system is proposed using a modified P+/N-well/P-sub structure that increases the capacity such as to reduce the number of self-reset operations and thus increase stability. An effective signal-to-noise ratio of 70 dB is achieved. Furthermore, an array of the modified photodiodes is developed and tested *in-vivo*.

An implantable fluorescence-imaging device is developed by Rebusi et al. and presented in the paper titled “Simultaneous CMOS-Based Imaging of Calcium Signaling of the Central Amygdala and the Dorsal Raphe Nucleus During Nociception in Freely Moving Mice” that has been used in animal experiments e.g., in the study of pain processing. Custom CMOS imager chips including a 120 by 40 pixel array are mounted on a needle-shape flexible PCB and equipped with optical filters and a blue-light micro-LED. Two such devices are implanted in a mouse to enable simultaneous recordings. Fluorescent imaging experiments are conducted and correlated with behavioral observations of a freely moving animal.

## Conclusion

Neuro-technology has become a keystone supporting scientific progress of modern neuroscience as well as the emergence of new therapeutic devices. Microelectronics is one major technology enabler of neuro-technology, alongside material science and bio-electronics, advanced signal and image processing. While microelectronics is based on mature fabrication technologies that scale down with time, significant progresses emerge at the circuit and system levels that improve the electrical characteristics of building blocks, which in turns enables the appearance of new application domains and therapeutic methods.

## Author Contributions

All authors listed have made a substantial, direct, and intellectual contribution to the work and approved it for publication.

## Conflict of Interest

The authors declare that the research was conducted in the absence of any commercial or financial relationships that could be construed as a potential conflict of interest.

## Publisher's Note

All claims expressed in this article are solely those of the authors and do not necessarily represent those of their affiliated organizations, or those of the publisher, the editors and the reviewers. Any product that may be evaluated in this article, or claim that may be made by its manufacturer, is not guaranteed or endorsed by the publisher.
